# Association between Seafood Intake and Cardiovascular Disease in South Korean Adults: A Community-Based Prospective Cohort Study

**DOI:** 10.3390/nu14224864

**Published:** 2022-11-17

**Authors:** Gyu-Hee Park, Jung-Hee Cho, Donglim Lee, Yangha Kim

**Affiliations:** 1 Department of Clinical Nutrition, Ewha Graduate School of Converging Clinical & Public Health, Seoul 03760, Republic of Korea; 2 Fisheries Policy Research Division, Korea Maritime Institute, Busan 49111, Republic of Korea; 3 Department of Nutritional Science and Food Management, Ewha Womans University, Seoul 03760, Republic of Korea; 4 Graduate Program in System Health Science and Engineering, Ewha Womans University, Seoul 03760, Republic of Korea

**Keywords:** cardiovascular disease, seafood intake, longitudinal study

## Abstract

Cardiovascular disease (CVD) is the most common non-communicable diseases causing 18.6 million deaths worldwide. Several studies have revealed that seafood consumption has a protective effect against CVD. This study investigated the correlation between CVD and seafood intake based on a 10-year follow-up of the Korean Genome and Epidemiology Study (KoGES). The study population, which included 6565 adults age, 55.65 (±8.68), was divided into seafood intake-based tertiles. CVD included myocardial infarction, coronary artery disease, congestive heart failure, cerebrovascular disease, and peripheral vascular disease. At baseline, participants with low seafood intake also had low eicosapentaenoic acid (EPA) and docosahexaenoic acid (DHA) intakes. Prospectively, hazard ratios (HRs) with 95% confidence intervals (CIs) for CVD were analyzed using Cox proportional hazards regression models. Seafood intake exhibited a significantly inverse relationship with the cumulative CVD incidence over 10 years regardless of sex (women: log-rank test *p* < 0.001 and men: log-rank test *p* < 0.0401). The longitudinal association of low seafood intake with the CVD risk was significantly stronger in female participants after adjusting for confounding variables (HR (95% confidence interval (CI)) = 0.718 (0.519–0.993) *p*-trend = 0.043). These results suggested that seafood consumption potentially ameliorates CVD risk in middle-aged adults.

## 1. Introduction

Cardiovascular disease (CVD) is the most common global non-communicable disease. Approximately 18.6 million people died from CVD in 2019 [1], accounting for one-third of global mortality [2]. According to the Centers for Disease Control and Prevention, CVD is the major cause of mortality in the United States (US), with one death occurring every 34 s and a mortality rate of one in five individuals [3]. In South Korea, CVD is the second leading cause of mortality [4]. The Korea Heart Disease Fact Sheet 2020, published by the South Korean Society of Cardiology reported that CVD-associated mortality and hospitalization rates had steadily increased in the preceding decade. A further increase in CVD-related indices is anticipated, and efforts focusing on prophylaxis and reducing the economic burden have been deemed essential [5]. Thus, CVD prevention has become the goal of public health care, both nationally and internationally.

Seafood has garnered considerable attention owing to its association with CVD. Numerous studies have demonstrated that fish intake may prevent CVD and reduce its risk [6,7,8,9,10,11]. A cross-sectional study reported that the consumption of copious amounts of seafood-based omega-3 poly-unsaturated fatty acids (N-3 PUFAs) among women correlated with a reduced CVD risk [9]. Several links between N-3 PUFAs and CVD risk have been suggested, including anti-inflammation, lipid-lowering effects [8], reduced platelet aggregation, and vasodilation [12].

N-3 PUFAs play a role in reducing inflammatory responses by engaging eicosapentaenoic acid (EPA-derived eicosanoids) to suppress its onset, thereby reducing CVD incidence [13,14]. Docosahexaenoic acid (DHA) also scales down the inflammatory response by decreasing neutrophil infiltration and anti-inflammatory mediator synthesis [13]. N-3 PUFA intake prevents the generation of triglycerides (TGs) in the liver via EPA and DHA [15]. Furthermore DHA appears to increase high-density lipoprotein cholesterol (HDL-C) and reduce low-density lipoprotein cholesterol (LDL-C) particle sizes [16]. EPA causes platelet-coagulation suppression [14] and promotes the expression and activation of endothelial nitric oxide synthase (eNOS), thereby enhancing endothelial function, leading to vascular expansion [16].

Although various studies have reported an association between seafood intake and reduced CVD risk, only a few have investigated this association via a prospective cohort study. This study aimed to determine the correlation between seafood intake and CVD using data from a 10-year follow-up community cohort of the South Korean Genome and Epidemiology Study (KoGES).

## 2. Materials and Methods

### 2.1. Participants

This study utilized data from the Ansan–Ansung community base prospective cohort of the KoGES. The geographic map of the participants is presented in Supplementary Figure S1 [17]. The KoGES is a large, prospective cohort study that aims to solve public health issues and prepare for personalized and preventive health care. It was initiated by the South Korean government (National Research Institute of Health, Centers for Disease Control and Prevention and the Ministry of Health and Welfare, South Korea). The KoGES investigates biannual repetitive surveys of participants recruited in 2001–2002 until follow-up [17].

The study population included adult men and women mean age 55.65 (±8.68), who participated in the second survey during the third KoGES phase that is, 2005–2006, and data obtained during the 10 years follow-up extending to 2015–2016 were utilized. Of 7515 participants (men: 3588 and women: 3927), we excluded 212 individuals who reported at least one CVD-related diagnosis, or history of treatment or drug administration in the basic survey. An additional 94 individuals were excluded because of a dietary history of unreliable energy intake (<500 kcal or ≥4000 kcal). Finally, 644 individuals with missing demographic (age and sex), lifestyle, and seafood intake data were also excluded. Therefore, the study eventually included 6565 participants (men: 3114 and women: 3451). At the end of the 10-year follow-up period, CVD occurred in 663 individuals (men: 315 and women: 348). This study was approved by the Institutional Review Board of Ewha Womans University (No. 202110-0022-01) and the subsequent study protocol complied with the principle of the 1975 Declaration of Helsinki and its 2008 revision.

### 2.2. Definition of CVD

According to the World Health Organization (WHO), CVD includes coronary artery or heart disease, cerebrovascular disease, peripheral artery disease, rheumatic heart disease, congenital heart disease, deep vein thrombosis, and pulmonary embolism [2]. In this study, CVD was defined based on the WHO definition and previous studies [18]. Thus, in the present investigation, CVD was confirmed by at least one “yes” response to a recent or previous diagnosis of and/or treatment or drug administration for the following: myocardial infarction, coronary artery disease, congestive heart failure, cerebrovascular disease, and peripheral vascular disease.

### 2.3. Seafood, EPA, and DHA Intakes

Dietary intake was analyzed using the semi-quantitative food frequency questionnaire (SQFFQ), with its validity and reproducibility were proven by Ahn et al. (2007) [19]. At baseline, participants responded to questions regarding the average intake frequency of 106 foods (rated on a nine-point scale: rarely, once a month, two to three times a month, once or twice a week, three to four times a week, five to six times a week, once a day, twice a day, or three times a day) and average per intake quantity (rated on a three-point scale: little, moderate, and high) for the year preceding the date of survey onset. The amount per intake (low, moderate, or high) was determined using participants‘ photographs of representative foods.

Seafood intake was analyzed using the participant’s SQFFQ data on 17 seafoods, including fish, shellfish, seaweed, and processed seafood (raw fish, blue-backed fish (mackerel, saury, mackerel, etc.), beltfish, eels, yellow croaker/sea bream/halibut, pollack/frozen pollack/dried pollack, squid/dried squid/octopus, anchovy or stir-fried anchovy, clam/whelk, oyster, shrimp, crab/marinated crab, laver, kelp/seaweed, tuna (canned), salted fish, and fish cake/crab stick). And seafood intake was computed as the sum of the intake calculated by multiplying the intake frequency and average intake of seafoods from the SQFFQ. Participants were divided into three seafood intake-based groups, namely, the first (T1, low), second (T2, intermediate), and third (T3, high) tertiles. The EPA and DHA content was acquired from the South Korea Standard Food Composition Table, 9th edition, published by the Rural Development Administration [20].

### 2.4. Measurements

Trained research staff took anthropometric measurements at each follow-up visit. The body mass index (BMI) was calculated as body weight (kg) divided by the square of the height (m^2^).

### 2.5. Covariates

At baseline, demographic and lifestyle surveys were used to extract information on age, sex, alcohol consumption status (never, past, or current), smoking status (current smoker or nonsmoker), regular exercise (yes or no), education level (elementary school or lower, middle school, high school, or college or higher), household income (<1 million Korean Won (KRW), 1–2 million KRW, 2–4 million KRW, or ≥4 million KRW). Intake of fruits and vegetables is known to have an independent effect on cardio-vascular disease and used as covariates.

### 2.6. Statistical Analysis

SAS 9.4 (SAS Institute Inc., Cary, NC, USA) was used for statistical treatment and analysis. To ensure a normal distribution, seafood intake was log-transformed before analysis. Continuous variables are expressed as the mean ± standard error (SE) and categorical variables as percentages (%). Analysis of variance and the chi-squared test were used to determine the mean and variation in the distribution of the general characteristics. A generalized linear model was used to analyze nutrient density according to seafood intake, and the *p*-trend was employed to identify significant trends. The cumulative CVD incidence was expressed on Kaplan–Meier curves, and the log-rank test was used to assess between-group variations. The follow-up years were presented as the average time of the period from baseline to occurrence of cardiovascular disease as the mean ± (SE). The hazard ratios (HRs) with 95% confidence intervals (CIs) of CVD based on seafood intake were determined using Cox proportional hazards regression models. Adjustments were made for potentially significant CVD confounders, including age, BMI, alcohol consumption, smoking status, exercise, education, household income, and vegetable and fruit intake. Statistical significance was set at *p* < 0.05.

## 3. Results

### 3.1. Baseline General Characteristics

Table 1 presents the participants’ general characteristics according to seafood intake. Seafood intake decreased with increasing age in both men and women. Men with low seafood intake exhibited lower levels of energy intake, BMI, alcohol consumption, regular exercise, education, and household income (*p* < 0.001). Women with low seafood intake had lower levels of alcohol consumption, smoking, regular exercise, education, and household income (*p* < 0.001), excluding BMI.

### 3.2. Food and Nutrient Consumption According to Seafood Intake

Table 2 presents the participants’ energy intake and nutrients density according to seafood intake. Caloric intake was adjusted to 1000 kcal to examine nutrient density. A low seafood intake indicated low energy intake and nutrient intake in all participants, with the exception of carbohydrates (*p*-trend < 0.001). Carbohydrate intake was significantly higher in participants with lower seafood intake (*p*-trend < 0.001).

### 3.3. EPA and DHA Consumption According to Seafood Intake

Table 3 shows the EPA and DHA values obtained from the overall food and seafood intake. Both men and women with low seafood intake had low EPA and DHA intakes (men: *p*-trend < 0.001 and women: *p*-trend < 0.001). The mean proportion of fish-based EPA intake (among all foods) was 94.64% in men and 95.46% in women, and that for DHA was 94.44% and 95.19% in men and women, respectively.

**Table 1:** General characteristics of male and female participants according to seafood-intake tertile.

Characteristics	Male				Female			
	Tertile 1	Tertile 2	Tertile 3	*p*-Value	Tertile 1	Tertile 2	Tertile 3	*p*-Value
	(*n* = 1038)	(*n* = 1038)	(*n* = 1038)		(*n* = 1148)	(*n* = 1152)	(*n* = 1151)	
Age (years)	57.53 ± 8.84	54.65 ± 8.41	53.45 ± 7.72	<0.0001	59.09 ± 8.83	55.61 ± 8.68	54.02 ± 8.14	<0.0001
BMI (kg/m^2^)	23.67 ± 2.96	24.32 ± 2.79	24.81 ± 2.78	<0.0001	24.78 ± 3.24	24.75 ± 3.14	24.82 ± 3.20	0.8804
Alcohol consumption status (*n*, %)								
Never	252 (24.28)	185 (17.82)	169 (16.28)	<0.0001	891 (77.61)	814 (70.66)	785 (68.20)	<0.0001
Past	95 (9.15)	109 (10.50)	68 (6.55)		16 (1.39)	24 (2.08)	15 (1.30)	
Current	691 (66.57)	744 (71.68)	801 (77.17)		241 (20.99)	314 (27.26)	351 (30.50)	
Smoking status (*n*, %)								
Nonsmoker	652 (62.81)	669 (64.45)	652 (62.81)	0.6705	1123 (97.57)	1133 (98.52)	1118 (97.22)	0.0472
Current smoker	386 (37.19)	369 (35.55)	386 (37.19)		28 (2.43)	17 (1.48)	32 (2.78)	
Regular exercise (*n*, %)	305 (29.38)	437 (42.10)	494 (47.59)	<0.0001	281 (24.41)	417 (36.20)	483 (41.96)	<0.0001
Education levels (*n*, %)								
Elementary or lower	352 (33.91)	184 (17.73)	137 (13.20)	<0.0001	779 (67.86)	518 (44.97)	376 (32.67)	<0.0001
Middle	241 (23.22)	226 (21.77)	191 (18.40)		184 (16.03)	230 (19.97)	266 (23.11)	
High	288 (27.75)	376 (36.22)	377 (36.32)		151 (13.15)	309 (26.82)	388 (33.71)	
College or higher	157 (15.13)	252 (24.28)	333 (32.08)		34 (2.96)	95 (8.25)	121 (10.51)	
Household income (*n*, %)								
<1 million KRW	404 (38.92)	210 (20.23)	165 (15.90)	<0.0001	685 (59.49)	429 (37.24)	335 (29.11)	<0.0001
1–2 million KRW	280 (26.97)	245 (23.60)	188 (18.11)		242 (21.08)	275 (23.87)	261 (22.68)	
2–4 million KRW	258 (24.86)	406 (39.11)	397 (38.25)		167 (14.55)	325 (28.35)	382 (33.19)	
>4 million KRW	96 (9.25)	177 (17.05)	288 (27.75)		56 (4.88)	123 (10.68)	173 (15.03)	

Values are expressed as the mean ± SE or percentages. *p*-values were obtained from analysis of variance (ANOVA) for continuous variables and chi-square tests for categorical variables. *p* < 0.05 was considered significant.

**Table 2:** Nutrient densities of participants according to seafood-intake tertile.

Variables	Male				Female			
	Tertile 1	Tertile 2	Tertile 3	*p*-Trend	Tertile 1	Tertile 2	Tertile 3	*p*-Trend
	(*n* = 1038)	(*n* = 1038)	(*n* = 1038)		(*n* = 1148)	(*n* = 1152)	(*n* = 1151)	
Energy intake (kcal)	1603.63 ± 388.94	1874.39 ± 427.26	2201.10 ± 542.86	<0.0001	1413.02 ± 365.67	1605.87 ± 410.33	1942.71 ± 516.97	<0.0001
Carbohydrate (g)	188.00 ± 13.02	178.37 ± 13.40	169.32 ± 14.76	<0.0001	194.01.35 ± 12.99	185.654 ± 13.23	173.98 ± 15.45	<0.0001
Protein (g)	28.86 ± 3.89	32.28 ± 4.09	36.55 ± 5.35	<0.0001	28.06 ± 4.15	31.54 ± 4.14	36.64 ± 6.07	<0.0001
Fat (g)	12.82 ± 5.01	16.08 ± 5.00	18.54 ± 5.1	<0.0001	10.58 ± 4.7196	13.37 ± 4.92	16.86 ± 5.35	<0.0001
Ca (mg)	183.41 ± 81.99	221.74 ± 83.07	252.84 ± 94.15	<0.0001	200.93 ± 97.57	254.23 ± 106.54	307.20 ± 117.56	<0.0001
P (mg)	441.8 ± 70.58	487.94 ± 69.68	537.56 ± 82.38	<0.0001	449.09 ± 80.12	500.98 ± 84.27	566.27 ± 102.95	<0.0001
Fe (mg)	4.58 ± 1.29	5.19 ± 1.2	5.8 ± 1.4	<0.0001	4.78 ± 1.39	5.51 ± 1.39	6.39 ± 1.80	<0.0001
K (mg)	1064.59 ± 363.84	1227.45 ± 329.93	1369.79 ± 362.59	<0.0001	1119.87 ± 403.33	1310.49 ± 397.95	1526.50 ± 470.32	<0.0001
Vit. A (mg)	218.77 ± 159.36	250.64 ± 135.33	280.15 ± 142.08	<0.0001	226.16 ± 171.36	263.95 ± 153.84	329.23 ± 189.66	<0.0001
Na (mg)	1471.74 ± 877.96	1555.04 ± 719.09	1571.52 ± 665.23	0.0028	1505.53 ± 910.47	1495.38 ± 763.70	1704.72 ± 849.08	<0.0001
Vit.B1 (mg)	0.53 ± 0.12	0.58 ± 0.11	0.61 ± 0.12	<0.0001	0.51 ± 0.11	0.55 ± 0.11	0.60 ± 0.12	<0.0001
Vit. B2 (mg)	0.41 ± 0.13	0.49 ± 0.12	0.55 ± 0.13	<0.0001	0.41 ± 0.14	0.5 ± 0.15	0.59 ± 0.17	<0.0001
Niacin (mg)	6.9 ± 1.27	7.77 ± 1.22	8.86 ± 1.58	<0.0001	6.68 ± 1.26	7.44 ± 1.29	8.66 ± 1.78	<0.0001
Vit. C (mg)	43.23 ± 24.7	51.13 ± 22.52	59.29 ± 24.79	<0.0001	51.72 ± 30.37	63.35 ± 30.33	74.33 ± 33.96	<0.0001
Zn (µg)	3.91 ± 0.52	4.23 ± 0.67	4.82 ± 1.56	<0.0001	3.94 ± 0.50	4.25 ± 0.64	4.63 ± 0.95	<0.0001
Vit. B6 (mg)	0.78 ± 0.19	0.85 ± 0.17	0.93 ± 0.17	<0.0001	0.81 ± 0.21	0.89 ± 0.19	1.00 ± 0.23	<0.0001
Folate (µg)	103.05 ± 49.88	114.39 ± 44.27	124.77 ± 43.17	<0.0001	113.51 ± 55.27	126.96 ± 50.90	146.67 ± 59.16	<0.0001
Retinol (µg)	21.95 ± 20.85	30.76 ± 20.27	39.63 ± 24.33	<0.0001	21.10 ± 22.09	32.20 ± 24.43	41.42 ± 25.01	<0.0001
Carotene (µg)	1145.36 ± 933.97	1275.00 ± 798.77	1389.63 ± 823.61	<0.0001	1202.02 ± 1007.64	1354.61 ± 894.62	1678.71 ± 1110.67	<0.0001
Ash (mg)	8.96 ± 6.47	9.31 ± 5.14	9.5 ± 4.6	0.0251	9.23 ± 7.04	9.34 ± 5.37	10.87 ± 6.5	<0.0001
Fiber (g)	3.00 ± 1.24	3.17 ± 1.06	3.27 ± 1.06	<0.0001	3.31 ± 1.30	3.49 ± 1.16	3.77 ± 1.32	<0.0001
Vit. E (mg)	3.58 ± 1.28	4.27 ± 1.25	4.92 ± 1.37	<0.0001	3.63 ± 1.35	4.42 ± 1.42	5.50 ± 1.86	<0.0001
Cholesterol (mg)	53.46 ± 44.2	80.41 ± 44.05	109.31 ± 43.64	<0.0001	44.67 ± 40.20	74.37 ± 43.28	110.48 ± 51.00	<0.0001

Values are expressed as the mean ± SE. *p*-trends was obtained using general linear model analysis. *p* < 0.05 was considered significant.

**Table 3:** EPA and DHA intakes of participants according to seafood-intake tertile.

Variables	Male					Female				
	Total	Tertile 1	Tertile 2	Tertile 3	*p*-Trend	Total	Tertile 1	Tertile 2	Tertile 3	*p*-Trend
	(*n* = 3114)	(*n* = 1038)	(*n* = 1038)	(*n* = 1038)		(*n* = 3451)	(*n* = 1148)	(*n* = 1152)	(*n* = 1151)	
Total food										
N-3 PUFA	2.104 ± 2.018	1.314 ± 1.432	2.008 ± 1.798	2.990 ± 2.349	<0.0001	1.931 ± 1.932	1.143 ± 1.357	1.785 ± 1.639	2.863 ± 2.272	<0.0001
EPA ¹ (g)	0.112 ± 0.099	0.036 ± 0.020	0.091 ± 0.030	0.207 ± 0.113	<0.0001	0.111 ± 0.119	0.033 ± 0.019	0.088 ± 0.031	0.212 ± 0.155	<0.0001
DHA ² (g)	0.198 ± 0.180	0.061 ± 0.038	0.165 ± 0.060	0.369 ± 0.209	<0.0001	0.187 ± 0.211	0.050 ± 0.033	0.146 ± 0.058	0.365 ± 0.277	<0.0001
Seafood										
N-3 PUFA	0.365 ± 0.339	0.111 ± 0.070	0.300 ± 0.111	0.684 ± 0.396	<0.0001	0.358 ± 0.397	0.098 ± 0.062	0.279 ± 0.108	0.696 ± 0.519	<0.0001
EPA ¹ (g)	0.106 ± 0.096	0.034 ± 0.02	0.088 ± 0.030	0.197 ± 0.110	<0.0001	0.106 ± 0.114	0.031 ± 0.018	0.084 ± 0.031	0.203 ± 0.148	<0.0001
DHA ² (g)	0.187 ± 0.178	0.054 ± 0.036	0.153 ± 0.058	0.355 ± 0.207	<0.0001	0.178 ± 0.208	0.045 ± 0.031	0.137 ± 0.056	0.352 ± 0.275	<0.0001

Values are expressed as the mean ± SE. *p*-trends were obtained using general linear model analysis. *p* < 0.05 was considered significant. ^1^ EPA is eicosapentaenoic acid. ^2^ DHA is docosahexaenoic acid.

### 3.4. Cumulative CVD Incidence According to Seafood Intake

**Figure 1:**
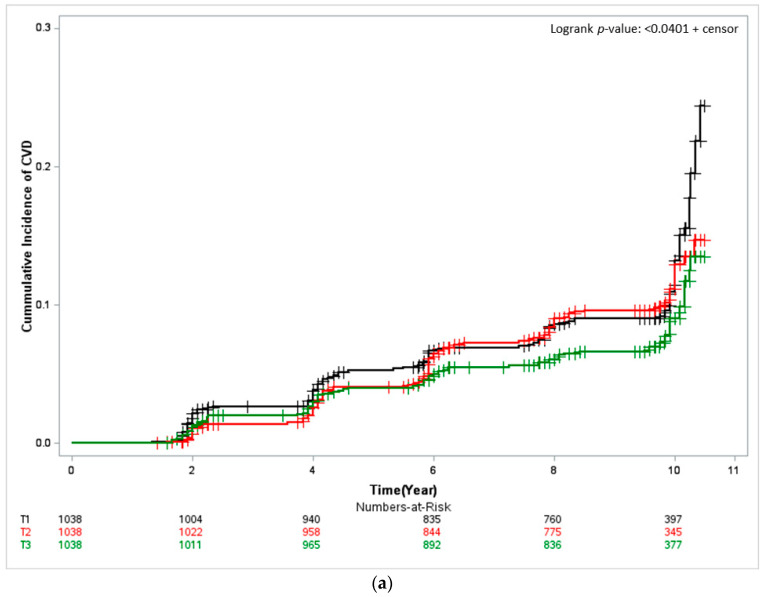

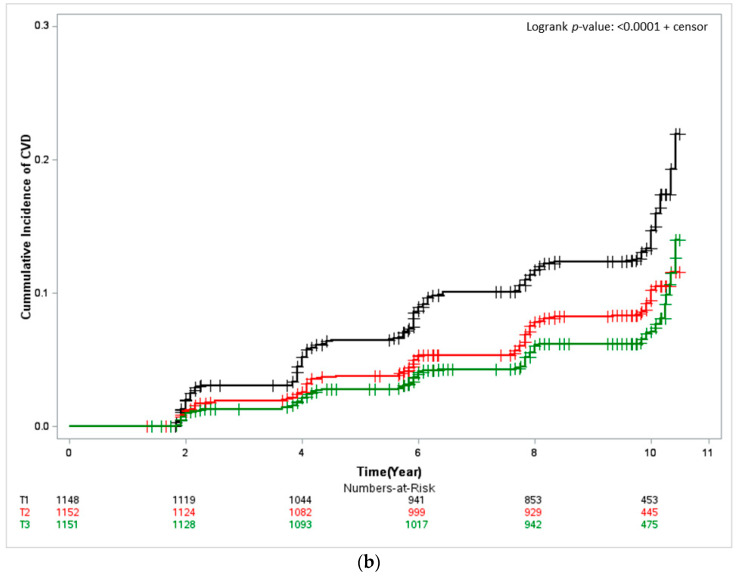
Kaplan–Meier curves showing the cardiovascular disease (CVD) cumulative incidence according to seafood-intake tertile. The log-rank test was used to determine risk differences among groups. (**a**) Male and (**b**) Female. T1, first tertile, black; T2, second tertile, red; T3, third tertile, green.

The Kaplan–Meier curves depicted in Figure 1 the cumulative CVD incidence, and describe results from approximately 10 years of follow-up. In both men and women, the cumulative CVD incidence was significantly low for the participants with the highest seafood intake (T3) (women: log-rank test *p* < 0.001 and men: log-rank test *p* < 0.0401). In men the cumulative CVD incidence per 1000 person-years was 13.67% (95% CI, 11.45–6.32) for T1, that is, the group of participants with the lowest seafood intake; 11.97% (95% CI, 9.92–14.45) for T2; and 9.45% (95% CI, 7.67–11.65) for T3. For women, it was and 15.97% (95% CI 13.69–18.65) for T1, 10.27% (95% CI 8.50–12.41) for T2, and 8.05% (95% CI 6.51–9.97) for T3.

### 3.5. Longitudinal Association of Seafood Intake with CVD Risk

The HRs for CVD incidence were analyzed using Cox proportional hazards regression models, after seafood intake-based follow-up (Table 4). In women, the CVD risk was higher for T1 (the group of participants with the lowest seafood intake) than for T3 (the group of participants with the highest seafood intake) (HR (95% CI) = 0.718 (0.519–0.993), *p* = 0.043) after adjusting for confounders, such as age, energy intake, alcohol consumption, smoking, exercise, education, household income, and vegetable and fruit intake. In contrast, no significant result was obtained for men (HR (95% CI) = 0.82 (0.580–1.159), *p*-trend = 0.2775).

## 4. Discussion

This study investigated whether CVD risk is influenced by seafood intake using a community-based prospective cohort. The results revealed that seafood intake was correlated with socioeconomic factors, but not with sex. Moreover, participants with high seafood intake exhibited higher nutrient, EPA, and DHA intake levels, whereas those with low seafood intake had a significantly higher carbohydrates intake. The 10-year follow-up study revealed that the cumulative CVD incidence was higher in participants with a low seafood intake, irrespective of sex. Women with low seafood intake demonstrated a significantly higher CVD risk than those with high seafood intake.

In this study, data from a 10-year follow-up showed that CVD risk was lower in participants with high seafood intake, and cumulative CVD analysis revealed that the time to CVD incidence increased with increasing seafood-intake tertile. A study that analyzed the association of CVD mortality with fish and N-3 PUFA intake using a community-based cohort of adult men and women in Japan found that the CVD-mortality risk decreased as fish and N-3 PUFA intake increased, with prolonged time to CVD [12]. A review of prospective studies on CVD and fish intake in the Mediterranean population also reported that CVD risk decreased with increasing fish consumption [21].

Participants with low seafood consumption exhibited low EPA and DHA intakes. Furthermore, EPA and DHA intakes via seafood (among all foods) were 94.64% and 94.44% in men and 95.46% and 95.19% in women, respectively. This implies that seafood is an important EPA and DHA source, as the HR of CVD incidence was low in participants with high seafood intake. Previous studies that investigated seafood intake in South Korean and Japanese cohorts demonstrated that N-3 PUFA, EPA, and DHA intakes were low in individuals with low seafood intake [22,23]. Seafood is considered an important dietary component mainly because it contains N-3 PUFAs, and the effects of EPA and DHA present in N-3 PUFA, such as enhanced endothelial function, anti-inflammation, and reduced blood viscosity, lower blood pressure and lipid concentration, reportedly leading to CVD-risk alleviation [24]. EPA and DHA intakes were shown to prevent CVD in a preliminary study of the Inuit population in Greenland, which consumed an N-3 PUFA-enriched diet [25]. Similarly, a study on CVD and N-3 PUFA intake in Japan demonstrated that the intake of PUFA containing EPA and DHA, lowered the risks of CVD incidence and mortality [26]. According to a study investigating the correlation between seafood and PUFA, seafood’s anti-inflammatory effects can potentially confer protective effects against atherosclerosis, plaque rupture, and CVD mortality, with a particular emphasis on its components, namely, EPA and DHA [27]. EPA promotes eNOS expression and activation, thereby enhancing endothelial function related to vascular relaxation and contraction [16]. Moreover, competitive absorption against arachidonic acid, an inducer of strong inflammatory responses, leads to platelet-coagulation suppression, and this anti-coagulant function reduces the degree of inflammation [14]. Inhibiting the expression of inflammatory genes responsible for signal relay contributes to CVD prevention by suppressing the onset of inflammation [28]. DHA prevents atherosclerosis, regulates cytokine-chemokine reduction and reactive oxygen species, and ameliorates the scale of the inflammatory response by decreasing neutrophil infiltration [28]. DHA exerts a protective effect against CVD via the synthesis of mediators, such as E- and D-series resolvins, DHA-derived protectin-D1 and maresin, that exert anti-inflammatory and immunoregulatory effects [13]. Both DHA and EPA reduce TG concentrations, probably by inhibiting VLDL-TG release and increasing TG clearance. DHA seems to increase HDL and LDL particle sizes by regulating cholesterol synthesis and lipid transfer between lipoproteins [16]. Therefore, EPA and DHA intake is potentially critical for CVD prevention, and as seafood is a major EPA and DHA source, its intake is likely reduce to CVD risk.

This study found that education levels and household income were lower in participants with low seafood intake possibly due to the high cost of fish consumption and limited knowledge regarding seafood’s value, resulting in low seafood intake among individuals of a relatively lower socioeconomic status. A study investigating the trends in seafood intake and its influencing factors in older South Korean adults found that individuals of low economic status consume expensive seafood less preferentially than they did other foods, while those with high education levels exhibited high seafood intake based on an awareness of seafood’s positive health effects and nutritional benefits [29]. According to a study on the preferential consumption of fish and its contributing factors in adults in Bangladesh, fish consumption was higher in individuals with a higher household income and education levels. Low fish consumption among low-income groups is reportedly attributed to financial limitations [30]. A study that analyzed the relationship between the economic status of American adults and obesity found that meat, fish, fresh vegetables and fruits were less consumed in low-income group. Because the cost per calorie of fish or fresh fruit/vegetables is higher [31].

Nutrient-density analysis according to seafood intake revealed that nutrient density was low in both male and female participants with low seafood intake. In contrast, carbohydrate intake was significantly higher in participants with low seafood intake. This observation is considered to be associated with socio-economic status, where the diet of individuals with low seafood intake and low socioeconomic status exhibited higher nutrient density via carbohydrate intake. A study that investigated food and nutrient intake with respect to income in a South Korean cohort found that energy and nutrient intakes were low in low-income individuals [32]. Considering the contribution of proteins, fats, and carbohydrates to total energy intake, carbohydrate intake was significantly higher in the low-income group [33]. A Japanese cohort study that analyzed socioeconomic status and high nutrient intake found a correlation between low household income and high cereal grain intake; the unbalanced consumption was reportedly due to the high intake of rice, a key starch source for individuals with low household income and low consumption of side dishes. This resulted from the limited affordability of the relatively high-cost ingredients in side dishes such as vegetables, fruits, and fish [34].

This study observed an inverse association between seafood intake and the HR of CVD in women only. This part is presumed to be caused by gender-specific metabolic differences in seafood and N-3 PUFAs intake in women and men. A study investigating the effects of N-3 PUFAs, EPA, and DHA, divided by gender, suggested that N-3 PUFAs were more effective in women and that differences occurred in the interaction of N-3PUFAs that inhibit sexual hormone and platelet activation. In men, EPA and DHA had differential effects, while women had both. In addition, both EPA and DHA have been reported that they are more effective against women than men [35]. A paper on sex differences in the content of N-3 PUFAs in tissues reported that women had higher circulating DHA concentrations than men. The proposed mechanisms responsible for sex differences observed in N-3 PUFAs reported differences in oxidation, adipose tissue composition and mobilization rates, and the effects of sex hormones on unsaturated enzymes and kidney enzymes involved in N-3 PUFAs synthesis [36]. This study suggests that sex differences should be considered in preventing or reducing CVD risk. Further studies on the mechanisms underlying sex differences in the association between seafood, N-3 PUFAs intake and CVD are warranted.

This study is highly significant because it entailed a longitudinal investigation of participants who were divided into three seafood intake based groups to verify the correlation of seafood consumption with the CVD risk over a 10 year period. Nevertheless, this study is limited in that dietary survey was implemented only by using a baseline survey, CVD-related blood parameter data were not researched and data on the occurrence of cardiovascular disease were self-reported.

## 5. Conclusions

This longitudinal study established an association between low seafood intake and low EPA and DHA consumption at baseline. This indicates that seafood is an important EPA and DHA source. The cumulative CVD incidence was higher in participants with low seafood intake. Furthermore, after 10 years follow-up, the HR of CVD was low in participants with high seafood intake. In conclusion, our findings suggest that CVD risk may be reduced by increasing seafood consumption in adults.

## Figures and Tables

**Table 4:** Hazard ratios (95% confidence intervals) of CVD incidence according to seafood-intake tertile.

Sex		Tertile 1	Tertile 2	Tertile 3	*p*-Value ^1^/ *p*-Trend
Male	Mean CVD incidence Period, years	8.53 ± 2.60	8.61 ± 2.44	8.87 ± 2.32	0.0048 ^1^
	Incident cases	121	107	87	
	Person-years	8850.25	8938.43	9201.95	
	Incidence rate per 1000 person-years (95% CI)	13.67% (11.45–16.32)	11.97% (9.92–14.45)	9.45 (7.67–11.65)	
	Crude model	1.0 (Ref)	0.894 (0.689–1.160)	0.704 (0.535–0.928)	0.0131
	Model 1	1.0 (Ref)	0.974 (0.741–1.280)	0.833 (0.601–1.155)	0.2911
	Model 2	1.0 (Ref)	0.976 (0.736–1.295)	0.82 (0.580–1.159)	0.2775
Female	Mean CVD incidence Period, years	8.62 ± 2.50	8.96 ± 2.20	9.06 ± 2.11	<0.0001 ^1^
	Incident cases	158	106	84	
	Person-years	9890.84	10,320.34	10,428.55	
	Incidence rate per 1000 person-years (95% CI)	15.97% (13.69–18.65)	10.27 (8.50–12.41)	8.05 (6.51–9.97)	
	Crude model	1.0 (Ref)	0.653 (0.511–0.836)	0.493 (0.379–0.643)	<0.0001
	Model 1	1.0 (Ref)	0.842 (0.652–1.088)	0.725 (0.533–0.985)	0.0355
	Model 2	1.0 (Ref)	0.849 (0.651–1.107)	0.718 (0.519–0.993)	0.0430

^1^*p*-values were obtained from ANOVA for continuous variables. Statistical significance was set at *p* < 0.05. Male model 1: adjusting for age, BMI, energy intake, exercise, alcohol consumption status, education level and household income. Male model 2: additionally adjusted for fruit and vegetable intake. Female model 1: adjusting for age, energy intake, exercise, alcohol consumption status smoking status, education level and household income. Female model 2: additionally adjusted for fruit and vegetable intake.

## Data Availability

Data are available from the Korean Genome and Epidemiology study (KoGES) conducted by the National Institute of Health, Centers for Disease Control and Prevention, Ministry for Health and Welfare, Republic of Korea.

## Supplementary Materials

The following supporting information can be downloaded at: https://www.mdpi.com/article/10.3390/nu14224864/s1, Figure S1: The geographic map of the participants.
