# Supplementary figures and images for: Association between Seafood Intake and Cardiovascular Disease in South Korean Adults: A Community-Based Prospective Cohort Study
Source: Nutrients. 2022 Nov 17;14(22):4864. doi: 10.3390/nu14224864 (PMC9695428; doi:10.3390/nu14224864)

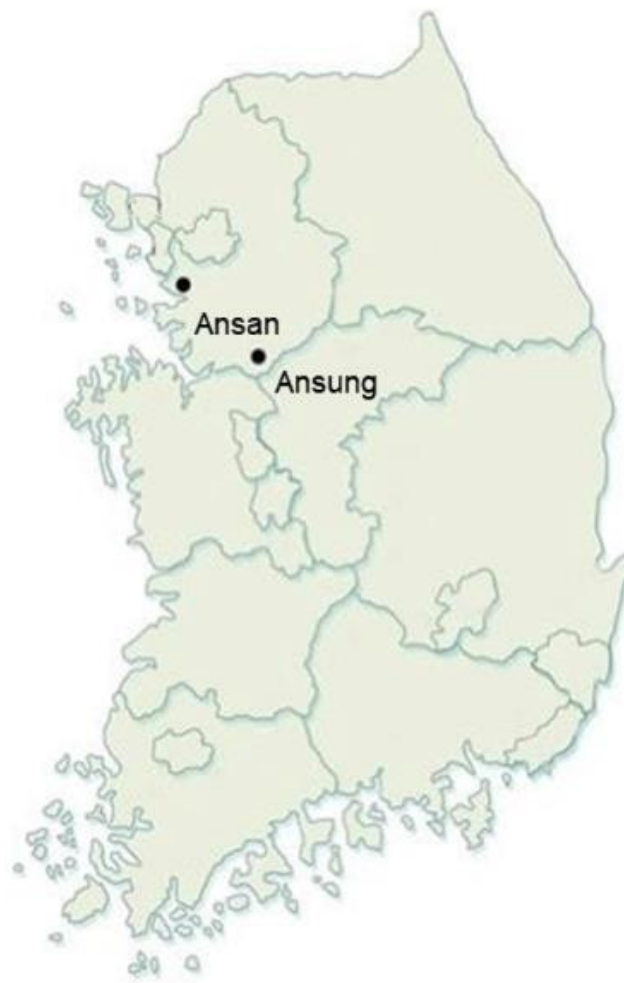

Supplementary Figure S1. The geographic map of the participants.

Supplement: Supplementary file 1 [file nutrients-14-04864-s001.zip › nutrients-2039494-supplementary.pdf]
